# Polymeric Micelles of Biodegradable Diblock Copolymers: Enhanced Encapsulation of Hydrophobic Drugs

**DOI:** 10.3390/ma11050688

**Published:** 2018-04-27

**Authors:** Yasser H. A. Hussein, Mohamed Youssry

**Affiliations:** Department of Chemistry and Earth Sciences, College of Arts and Sciences, Qatar University, Doha 2713, Qatar

**Keywords:** diblock copolymers, polymeric micelles, drug encapsulation, physicochemical properties, biodegradable/biocompatible copolymers

## Abstract

Polymeric micelles are potentially efficient in encapsulating and performing the controlled release of various hydrophobic drug molecules. Understanding the fundamental physicochemical properties behind drug–polymer systems in terms of interaction strength and compatibility, drug partition coefficient (preferential solubilization), micelle size, morphology, etc., encourages the formulation of polymeric nanocarriers with enhanced drug encapsulating capacity, prolonged circulation time, and stability in the human body. In this review, we systematically address some open issues which are considered to be obstacles inhibiting the commercial availability of polymer-based therapeutics, such as the enhancement of encapsulation capacity by finding better drug–polymer compatibility, the drug-release kinetics and mechanisms under chemical and mechanical conditions simulating to physiological conditions, and the role of preparation methods and solvents on the overall performance of micelles.

## 1. Introduction

Block copolymers are a fascinating class of polymeric materials that consist of two or more covalently bonded blocks forming a variety of architectures (e.g., linear diblock and triblock copolymers, star block copolymers, and miktoarm star copolymers) [1]. They are commonly used in many biomedical applications, such as scaffolds for tissue engineering [2] and anticancer drug nanocarriers [3]. One special class of block copolymers are the so-called amphiphilic block copolymers. By definition, they comprise hydrophilic (water-loving) and hydrophobic (water-hating) polymer blocks. As the most common solvent, water is a selective solvent for one block (i.e., the hydrophilic block). Above a certain concentration—the so-called critical micelle concentration (CMC)—the hydrophobic effect [4] drives the block copolymers in an aqueous environment to self-assemble, producing supramolecular aggregates with various morphologies, such as spherical and cylindrical micelles and vesicles. Such structures of nanometeric- to micrometric-size scales are difficult to be obtained by conventional chemical reactions. The final size and morphology of the aggregates are an expression of an optimum thermodynamic state, in which the sum of such factors as chain stretching, interfacial tension, and repulsive interactions between head groups (hydrophilic blocks) are minimized [5]. The application of theoretical models to predict the evolution of the morphology of aggregates in block copolymers systems is experimentally restricted [6]. Instead, an empirical law for neutral and flexible copolymers in water has been proposed by Disher and Eisenberg: vesicles are formed when f (the mass of the hydrophilic blocks to the total mass of the copolymer) is equal to 35 ± 10%, block copolymers with f > 45% are expected to form spherical micelles, and those with f < 25% are expected to self-assemble into inverted structures [7]. Among these morphologies, spherical micelles (namely, polymeric micelles) will be the aim of the current research project due to their peculiar structure and simplicity.

**Figure 1:**
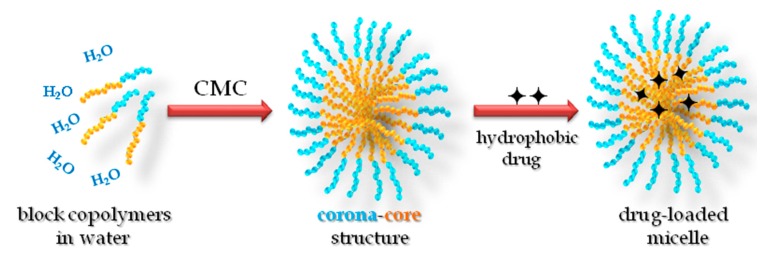
Schematic representation for the micellization of diblock copolymers and drug encapsulation in polymeric micelle. CMC: critical micelle concentration.

The polymeric micelle consists of two distinct regions—an interior region of hydrophobic polymer chains (the core region) and an outer region of well-solvated hydrophilic polymer chains (the corona or shell region; Figure 1), which imparts colloidal stability [8,9]. Block copolymers can be designed to exhibit very low CMC (0.1–1 μM [10,11]) compared with low-molecular-weight surfactants (0.1–1 mM). The CMC provides an indication of the thermodynamic stability of the micelles, because it expresses the minimum concentration of polymers at which the micelles remain self-assembled (i.e., the stability) [12]. It is affected by many factors, including the properties of the core-forming blocks, such as hydrophobicity, the glass transition temperature (T_g_), the degree of crystallinity, and the hydrophilic/hydrophobic length blocks ratio [13]. Other factors are thoroughly reviewed elsewhere [14]. Above the CMC, polymeric micelles are in equilibrium with the unimers, in a situation qualitatively analogous to classical low-molecular-weight surfactants. However, polymeric micelles are assumed to have higher thermodynamic and kinetic stability (slower dissociation rate into unimers) than surfactant micelles due to the integrated molecular effect and the entangling of the core-forming blocks [15]. Generally, the size of polymeric micelles is of the order of tens to hundreds of nanometers [1].

The peculiar corona-core structure and properties of polymeric micelles uniquely give them the ability to enhance the aqueous solubility of water-insoluble hydrophobic substances. The solubility enhancement arises from the fact that the micellar cores can serve as compatible microenvironments for water-insoluble solute molecules. This phenomenon of enhanced solubility is referred to as “solubilization” [16,17,18]. Ganesh and Nagarajan have developed the theory of solubilization based on the thermodynamic considerations of block copolymers, with the assumption that the micelles containing the solubilizate can be considered as a pseudo-phase in equilibrium with the solubilizate and the block copolymer molecules in a solution [17]. Accordingly, explicit calculations of the solubilization capacity of the micelles, the dimensions of the hydrophobic core swollen by the solubilizate, and the hydrophilic shell, as well as the change in aggregation number N_agg_ and in the CMC for a series of diblock poly(ethylene oxide)-poly(propylene oxide) PEO-PPO and triblock poly(ethylene oxide)-poly(propylene oxide)-poly(ethylene oxide) PEO-PPO-PEO copolymers have been made. A reasonable agreement has been found between the experimental and theoretical solubilization capacity values for PEO-PPO diblock copolymers in water and benzene as solubilizate. It could be shown that the volume fraction of solubilizate (φ) in the micellar core scales with the degree of polymerization (number of monomers per block) of both the core (N_B_) and corona (N_A_) blocks is according to the following relation: φ~N_B_^−0.17^ N_A_^−0.017^ [17]. The selective solubilization for a particular component in mixed solubilizates confirms the fact that the solubilization capacity is mainly controlled by the Flory–Huggins interaction parameter (χ), characterizing the interaction between the solubilizate and the core-forming blocks.

The polymeric micelle can efficiently accommodate the hydrophobic drug simply via physical entrapping (solubilization) in its hydrophobic core so that some advantages can be gained: (i) elimination of drug side effects; (ii) protection of drug molecules against possible degradation in particular media (pH, temperature); (iii) increasing the aqueous solubility of hydrophobic insoluble drugs; and (iv) control the drug release rate. Furthermore, recent advances in synthetic chemistry have enabled chemical conjugation of drugs in the micelle core [13,15,19], as well as designing smart polymeric micelles with functions such as molecule-specific targeting [20,21,22,23] and stimuli-responsive drug release [3,24,25,26,27]. 

Biological and physicochemical criteria should be considered in order to formulate a drug nanocarrier satisfying all the aforementioned advantages. Table 1 summarizes some biological requirements for and corresponding features of polymeric micelles that should be satisfied for the polymeric micelles to be considered an efficient drug delivery system. In the next paragraph, the biological requirements are briefly discussed. Moreover, a more detailed discussion of the physicochemical properties of polymeric micelles and their impact on the optimization of a drug delivery system will be extensively demonstrated in Section 2.

**Figure 2:**
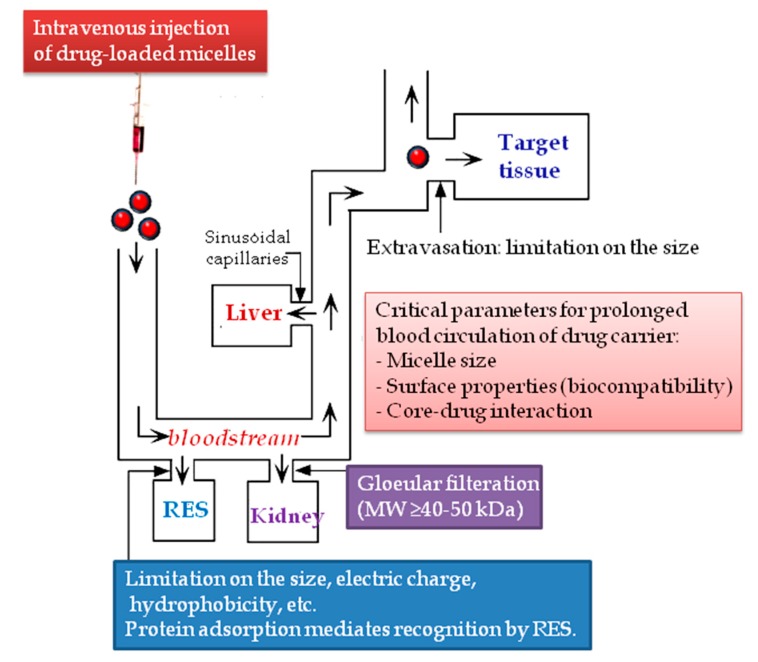
Itinerary of a drug carrier after intravenous administration. Adapted with permission from [13]. RES: reticuloendothelial system.

Upon intravenous injection of polymeric micelles in the human body, sever dilution (sinking) necessarily affects the micelles’ stability so that micelles may disassemble into unimers. However, the very low CMC of copolymers somewhat lends a kind of stability that leads to the overcoming of the sinking condition [29]. Furthermore, the disassembly of micelles is mentioned to be advantageous because this will facilitate elimination of the copolymer material from the body via the filtration in the kidneys. The main obstacles to the circulation of polymeric micelles are the filtration in the kidney and recognition by the reticuloendothelial system (RES) located in the liver, spleen, and lung (Figure 2) [30]. This can be overcome when corona-forming blocks are highly biocompatible [31] and the total molecular weight of the block copolymer is higher than 42–50 kDa—the molecular weight threshold for water-soluble synthetic polymers to be filtered or recognized [32].

The micelle size should be less than 100–150 nm, because larger micelles may be susceptible to recognition and removal by the RES [13]. Also, it appears that smaller micelles might show a high accessibility to tumor tissues [33,34]. For long-circulating drug delivery in the bloodstream, size-sieving is another challenge rather than recognition by the RES. Therefore, preparation of highly monodisperse micelles is strongly recommended to avoid size-sieving in the body [35].

Nontoxic biocompatible hydrophilic blocks and biodegradable hydrophobic blocks will determine how much the highly monodisperse polymeric micelles are stable with low CMC and nanometeric size to be accepted as drug carriers. The total molecular weight of the block copolymer, its polydispersity, the lengths and nature of the hydrophilic and the hydrophobic blocks and the ratio between them—hydrophilic-lipophilic balance (HLB)—are the most important physicochemical parameters that govern the micelles’ stability, size, and polydispersity. The properties of the hydrophobic blocks, including their polarity, hydrophobicity, degree of crystallinity, glass transition temperature (T_g_), the drug–core compatibility (in terms of Flory–Huggins interaction parameter, χ), and the drug/polymer weight ratio will play the most crucial role in determining the drug-loading efficiency and kinetics of release. Furthermore, the micelle preparation method is another influential factor in determining the overall micelle properties, including size, polydispersity, and loading efficiency. All these criteria and their impact on the performance of polymeric micelles are depicted in Table 2 and discussed in detail in Section 2.

Due to the lack of a common polymeric micelle that gives a high loading efficiency for many drugs, the optimization of polymeric micelles by modulating the abovementioned factors is highly desired. The development of biocompatible and biodegradable drug carriers, which possess small particle size, high loading efficiency, extended circulation time, and the ability to accumulate in required pathological sites in the body, for the delivery of poorly soluble pharmaceuticals still has many unresolved issues. Systematic studies based on prior knowledge of the physicochemical characteristics of particular block copolymers and drug molecules are still scarce. 

Nanostructured polymeric micelles and other nanoparticles create so-called nanomedicine, opening the door for diagnosis and management of life-threatening diseases, such as cancer. Nanomedicine is more of a new chemical entity than the conventional counterparts in terms of entrapment, solubilization, or controlled drug release and targeting without resorting to chemical conjugation. After the first pioneer article by Bader et al. [36], the study of polymeric micelle drug carrier systems started [37,38,39], and these carrier systems were recognized as one of the most potent drug carrier types in the 1990s [10,12,29,40,41,42,43,44,45,46,47,48,49,50] after the leading trials for the enhancement in in vivo pharmacological activities [39,51] and targeting [40,52] of drugs through the use of polymeric micelles. Then, in the 2000s, several significant, related physicochemical and clinical studies got underway [11,13,53,54,55,56,57,58,59,60,61,62,63,64,65,66,67,68,69,70,71,72]. Recently, many review articles summarizing the latest development in this field have appeared (see e.g., [22,73,74,75,76,77,78]).

Numerous types of biodegradable and synthetic block copolymers with different architectures (diblock, triblock, and grafted copolymers) and physical natures (charged and neutral) have been used to prepare diverse nanostructures, such as vesicles [7,79,80,81] and spherical and rodlike [82] micelles for drug delivery and targeting (passive and active [83]) purposes. Diblock copolymers are characterized by their lower CMC [18] and hence, more thermodynamic stability [29], as well as higher drug-loading capacity [18], than triblock copolymers at the same molecular weight or HLB. Neutrally charged spherical micelles, with their relatively smaller size and polydispersity compared with vesicles, are considered potent drug delivery systems that will not be easily recognized by the RES. Therefore, the current study will focus on the investigation of the physicochemical properties of spherical polymeric micelles formed from neutral biodegradable diblock copolymers. In addition, the physical incorporation of drug will be considered to avoid the pH effect on the drug–core bonds in the case of conjugated drugs. 

So far, polymeric micelles intended for biomedical use have been prepared from a variety of amphiphilic block copolymers, including poly(ethylene glycol)-poly(γ-benzyl l-glutamate) PEG-PBLA [10,41,43,44,54,55,68,84,85,86,87], poly(ethylene glycol)-poly(d,l-lactic acid) PEG-PDLLA [11,45,72,86,87,88,89,90], poly(ethylene glycol)-poly(l-lactic acid) PEG-PLLA [91,92,93], poly(ethylene glycol)-poly(ε-caprolactone) PEG-PCL [29,46,47,59,61,63,66,67,70,72,87,93,94,95,96,97,98,99,100], poly(ethylene glycol)-poly(d,l-lactide-co-glycolide) PEG-PLGA [30,101,102], poly(ethylene glycol)-poly(γ-benzyl l-glutamate) PEG-PBLG [103,104], poly(ethylene glycol)-poly(β-benzyl l-aspartate) PEG-PBLA [12,40,43,44,85,105], poly(ethylene glycol)-poly(α-benzyl carboxylate-ε-caprolactone) PEG-PBCL [67,93,100], and poly(ethylene glycol)-poly(δ-valerolactone) PEG-PVL [106,107]. 

## 2. Characteristics of Diblock Copolymers 

The unified aims of the aforementioned research articles were the enhanced solubilization of poorly water-soluble drug molecules in the core of polymeric micelles achieving, to some extent, controlled drug release in tandem with considerable micellar stability in the sinking (physiological) conditions. We may divide these aims into the following separate points to better understand the factors through which they can be achieved: (i) the micellar stability in outdoor and physiological environments is strongly dependent on the micelle size and the CMC, which in turn is influenced by the physicochemical parameters and characteristics of the polymer (its molecular weight, HLB, and its constituent hydrophobic (its length, hydrophobicity, degree of crystallinity, and polarity) and hydrophilic blocks); (ii) the enhanced solubilization of drugs in the micellar core seems to be strongly dependent on the micelle size and size distribution (polydispersity), both core blocks (hydrophobicity and polarity), the drug molecule (molar volume *v*_m_ and partition coefficient K_V_), and the interaction strength between the core blocks and drug molecules (χ); (iii) this interaction parameter together with the core diameter will reflect the speed of the drug release rate; and (iv) the micelle preparation and drug incorporation protocols, including the used solvent, the complementary sonication, centrifugation, etc., are very crucial in determining the properties and performance of free and loaded micelles. The comparisons between different results introduced by various research groups are depicted in Table 3. In the next paragraphs, detailed descriptions for each parameter and its impact on the micellar stability, enhanced drug solubility (loading efficiency), and release rate [45,66,86,107] is discussed. 

### 2.1. The Molecular Weight and Polydispersity of the Polymer

The molecular weight of the block copolymer should exceed a threshold value (42–50 kDa for water-soluble synthetic polymers [32]) to avoid a possible glomerular (renal) filtration in the human body. Rationally, the increase in the molecular weight necessarily decreases the CMC and increases the micelle size, resulting in the increasing of the micelle core and then the drug-loading capacity [45,66,86,107]. Shin et al. reported a monotonic increase in the micelle size with the molecular weight until a threshold, beyond which no micellization occurs because of too long hydrophobic blocks [47]. Another important parameter related to the block copolymer is its polydispersity. The narrower size distribution of polymeric micelles (i.e., monodisperse) is highly desired and can be obtained by using block copolymers with low polydispersity [35,47] in order to avoid size-sieving in the bloodstream.

### 2.2. The Critical Micelle Concentration

The CMC provides an indication of the thermodynamic stability of the micelles or the minimum concentration at which these nanoparticles will stay self-assembled. Possible micelle dissociation upon dilution certainly has an important influence on the drug-delivering capacity of polymer micelles composed of amphiphilic block copolymers. Therefore, a lowered CMC is favorable for drug retention in the micelle in vivo under considerable dilution [35]. It has been shown that a decrease in the CMC results in an increase in the total number of copolymer molecules participating in the formation of micelles, thus increasing the number of micelles in the solution available for the solubilization of the drug molecules [112]. However, other groups have noted that as the hydrophobic block length of a series of copolymers increases, the aggregation number of the micelles correspondingly increases, resulting in a larger core volume and providing more space for the solubilization of greater amounts of solute [113]. It is stated that the CMC decreases as the hydrophobic length increases [47,60,103,107]. However, this fact is not correct without considering the hydrophobic/hydrophilic ratio. Indeed, the CMC is found to decrease as the hydrophobic/hydrophilic ratio increases, producing more stable micelles [12,35,47,66,106]. Yasugi et al. reported a CMC range from 2.5 to 4.5 mg/L as the PDLLA/PEG weight ratio was decreased from 1.3 to 0.5, respectively [35]. In contrast, Letchfordd et al. have revealed recently that the CMC is independent of the PCL/MPEG ratio; instead, it is more sensitive to the PCL length [66]. In the two studies, the authors compared the CMC of block copolymers with different hydrophilic lengths. A careful accounting for the dependency of CMC on the hydrophobic/hydrophilic weight ratio should be done at a fixed hydrophilic length, otherwise conflicting conclusions may arise. 

Table 4 summarizes the effect of molecular weight (MW), the hydrophobic length, and hydrophobic/hydrophilic ratios of different diblock copolymers on the CMC. It is obvious that at a constant hydrophilic block length, the CMC decreases as the hydrophobic/hydrophilic ratio (or hydrophobic length) increases [12,35,61,106]. From the data depicted in Table 4, we may account for a decrease in the CMC of different block copolymers (at a nearly constant hydrophobic/hydrophilic ratio) as follows: PEG-PCL ≅ PEG-PDLLA < PEG-PBLA < PEG-PVL.

### 2.3. The Hydrophilic (Corona-Forming) Blocks

Among numerous biocompatible polymers, poly(ethylene glycol) (PEG) is the most commonly used corona-forming block, due to the high flexibility of its structure, high degree of hydration, nontoxicity, and weak immunogenicity, and as such, it has been approved by the Food and Drug Administration (FDA) [114]. Most of the previous studies have shown that PEG has been the preferred choice of hydrophilic block that imparts colloidal stability for the polymeric micelle. It should be mentioned that PEG and PEO are different nomenclatures for the same compound, and some studies have used its analogous, methoxy poly(ethylene glycol) (MPEG) as a hydrophilic block [45,47,61,66,96,99]. PEG chains are devoid of pendant sites that could be used to conjugate various functional groups for active targeting [80,106,115,116,117,118]. Due to steric repulsion [119], the outer PEG shell of the micelle inhibits the surface adsorption of proteins and other biological components in the bloodstream so that there is no recognition by the RES (such as the liver, kidney, or spleen) [114], achieving higher circulation half-time in the body [101,120,121] and having protective effect during prolonged circulation [33]. 

Whether the hydrophobic length or the hydrophobic/hydrophilic ratio is the key determining factor for the micelle size is another controversial issue. Yasugi et al. reported a reduction in the micelle size from 154 nm to ca. 30 nm as the PDLLA/PEG ratio increases from 0.3 to 1.3 [35]. Nevertheless, the correlation between micelle size and PDLLA/PEG ratio is nonmonotonic, and copolymer composition should be tuned to achieve optimum size and polydispersity of micelles, as well as loading efficiency. However, Richter et al. reported recently an optimum hydrophobic/hydrophilic ratio of 1, at which higher loading efficiency and micelle stability could be attained for PEG-PCL and PEG-PDLLA micelles [72]. In Table 3, it is somewhat difficult to account for whether the dependency of micelle size is on hydrophobic block length or the ratio between hydrophobic and hydrophilic blocks. Nevertheless, attention should be paid to the effect of the total MW of the copolymer and the preparation method (which will be discussed in detail). However, a rough conclusion may be stated that, ignoring the preparation method, the micelle size increases as the hydrophobic/hydrophilic ratio increases, and range of 0.7–1.0 seems to be optimum for producing a micelle size of less than 100 nm.

To obtain polymeric micelles that exhibit stable circulation in the bloodstream, the hydrophilic corona-forming block of PEG needs to be regulated at a MW of 5 to 12 kDa, and the length of it should preferably be greater than that of the core-forming block [42]. Increasing the amount of PEG reduces the polydispersity of the system, and a more hydrophobic and complex core would demand a higher chain length and density of PEG (more bound water in the surrounding medium) to obtain the optimal colloidal steric stabilization [88]. However, there exists an optimum PEG length at which higher loading efficiency and micellar stability could be achieved. In general, an increase in the corona block will result in an increase in the CMC [106], followed by a decrease in the aggregation number (N_agg_), and ultimately, smaller micelles [112] possessing water molecules at the corona [122] will be formed. Consequently, a decrease in the partition coefficient of the drug occurs and hence, a reduced loading efficiency results. The effect of PEG length (or MW) on the loading efficiency should be accounted for, considering the hydrophobic/hydrophilic ratio. Zhang et al. reported a decrease in the Taxol-loading efficiency upon increasing the MW of MPEG in MPEG-PDLLA micelles [45]. However, that is not the case if they take into account the PDLLA/MPEG ratio, where the micelle of MPEG_5000_-PDLLA_2000_ (PDLLA/MPEG = 0.4) has lower loading efficiency (10%) than the micelles of both MPEG2000-PDLLA_1400_ (PDLLA/MPEG = 0.7) and MPEG_2000_-PDLLA_2000_ (PDLLA/MPEG = 0.1) (25%). This behavior is not due to the longer MPEG but due to the decreasing in PDLLA/MPEG ratio. Richter confirmed this trend recently and found that the Saglopine-loading efficiency in PEG-PCL micelles increased as the PCL/PEG ratio was increased [72].

### 2.4. The Hydrophobic (Core-Forming) Blocks

The nature and physicochemical properties of the core-forming (hydrophobic) blocks play a crucial role in determining a micelle’s characteristics and performance in loading and release profiles. To be considered in drug formulations, the core-forming blocks should have nontoxic and biodegradable natures with defined degradation rates, such as polycaprolactones (PCL), polylactic acids (PLA and PDLLA), and polyamino acids (PBLA and PGLA). As the hydrophobicity of the core-forming blocks increases, the release rate decreases [64,65], and the thermodynamic stability of micelles is enhanced [29]. At the same molecular weight, highly hydrophobic PVL or PCL enhance the thermodynamic stability [29] and decrease the drug release rate [123] much more than relatively less hydrophobic PDLLA. Yu et al. increased the hydrophobicity of PBLA blocks by hydrolysis or ester exchange reaction in order to increase the solubilization of a drug in the core of micelles [84]. Replacing the aromatic moiety of PBLA with aliphatic ones through polymer-analogous reactions resulted in a decreasing of the polarity of the micelle cores and then a higher incorporation of the drug [54].

Again, many researchers have reported that the length of core-forming blocks has an appreciable effect on the micelle size and loading efficiency [48,66,96,98]. As the hydrophobic block length increases, the aggregation number (N_agg_) of the micelle increases, resulting in a larger core, which allows for a higher loading efficiency. In addition, longer hydrophobic blocks result in a decrease in micelle polydispersity. As the PCL length increases, the size of the MPEG-PCL micelle increases; however, a negligible change in the Doxorubicin loading efficiency (3–4%) was noticed. The authors attributed this poor loading efficiency of Doxorubicin to the weak hydrophobicity of Doxorubicin (because it contains –OH and –NH_2_ groups), to the hydrogen-bonding interaction between PCL and Doxorubicin, and to the increased crystallinity of PCL as a consequence of increasing the PCL length. The same authors reported a conflicting conclusion for the effect of PCL length on the Paclitaxel-loading efficiency in MPEG-PCL [96], where they reported that as the PCL length increased the Paclitaxel-loading efficiency increased. In addition, Soo et al. [98] attributed the increase in loading efficiency of PEG_1980_-PCL_x_ micelles from 10–90%, to the increasing of PCL length x from 1368 to 17,328 Da, respectively. This is not always the case, and careful investigation of the effect of hydrophobic length should not be studied alone; the hydrophobic/hydrophilic ratio should be taken into consideration when assessing the effect of hydrophobic length on the loading efficiency, as discussed above. Aliabadi et al. found a nonmonotonic effect of PCL length on the Cyclosporin A (CsA)-loading efficiency in MPEG-PCL and an optimum polymer composition [97]. Richter et al. concluded that the increased Saglopine-loading efficiency is more attributed to increasing the PCL/PEG ratio than increasing the length of PCL alone [72]. More examples for different block copolymers are demonstrated in Table 3.

### 2.5. The Crystallinity of Core-Forming Blocks

The glass transition temperature (T_g_) of the hydrophobic segment has also been shown to have a direct effect on the CMC, micellar stability, and drug release rate [124]. At temperatures above T_g_, the CMC value increased with the temperature according to the following equation, ΔG_0_ ∼ RTln(CMC), where G_0_ and R are the Gibbs standard free energy and the universal gas constant, respectively, and the micelle core is in a liquid-like state. In contrast, an almost constant CMC is observed, regardless of the temperature change, below the T_g_ [11], where the micelle is called a “frozen micelle” [29]. This result is associated with a gradual increase in the chain mobility of the hydrophobic segment in the core of the micelles above T_g_, where the increased core fluidity (i.e., is less frozen) imparts a lower stability of polymeric micelles [125], because unconstrained (free) molecular motions of the hydrophobic chains in the core account for lower kinetic stability upon dilution (sinking conditions) [29]. This confirms the fact that the crystallinity of the core-forming blocks significantly contributes to the micellar stability and may confer greater drug retention properties by decreasing the rate of drug diffusion from the micellar core [126,127], because the drug molecules diffuse more slowly from a frozen (glassy) core than liquid-like one [29]. 

The semicrystalline nature of materials, such as PCL and PVL, may result in micelles with enhanced kinetic stability when compared with micelles formed from copolymers with the amorphous polymer PDLLA as the core-forming block [29]. The degree of crystallinity and T_g_ values for some hydrophobic blocks are depicted in Table 5. For PEG-PDLLA polymeric micelles, an increase in CMC has been reported above 42 °C (T_g_ of PDLLA) and a constant CMC value (0.6 μM) below the T_g_ regardless of the temperature [11]. It is also stated that as the hydrophobic block length increases, the crystallinity of polymer increases [47]. For the same polymeric micelle system, Burt and coworkers reported that as the CMC increases, the core fluidity increases (i.e., is less frozen) imparting a lower kinetic stability of PEG-PDLLA polymeric micelles [125]. The mobility of the core-forming segment is also related to the exchange behavior of the constituent block copolymers between the micelles [13]. The chain exchange rates between the PEG-PDDLA micelles were found to be accelerated by increasing the temperature from 25 to 40 °C. The frequency of the chain exchange rate may correlate with the possible interaction of the block copolymers with biological components, including proteins and cellular membranes. T_g_ has an appreciable effect on the solubilization capacity, because the partition coefficient increases with longer hydrophobic blocks, and then, the solubilization capacity increases [59]. 

## 3. Characteristics of Polymeric Micelles

### 3.1. The Drug Partition Coefficient

The partition coefficient is a convenient way to express the affinity of the drug for the micelle core or for the external environment. It is simply defined as the ratio between the drug concentration in the micelle core to its concentration in the external aqueous solution [113]. The partition coefficient (KV) of a drug molecule can be calculated using the following equation [94]:

(1) $$\frac{{\left[drug\right]}_{micelle}}{{\left[drug\right]}_{aqueous}}\;=\;K_{V}X_{cb}\frac{C}{\rho }$$

where [*drug*]*_micelle_* and [*drug*]*_aqueous_* are the drug concentration in the micelle and in the aqueous medium, respectively, *X_cb_* is the mole fraction of core-forming blocks in the copolymer, *C* is the concentration of the copolymer, and *ρ* is the bulk density of the core-forming blocks. As the core-forming block lengths increase, the partition coefficient was found to increase and hence the drug-loading efficiency increased [29,59,66]. Increasing the temperature from 20 to 37 °C has been found to increase the partition coefficient as a result of the decrease in the degree of hydration of the core-forming block at the higher temperature [46].

Although the partition coefficient was found to increase as the polarity of the core-forming blocks decreases [29,59], Letchfordd et al. [66] reported that this fact does not always hold and the partition coefficient is very sensitive to the compatibility between the drug molecules and the micelle core. They eventually concluded that the drug-loading efficiency is not related to the partition coefficient, but is better described by the Flory–Huggins interaction parameter, which accounts for the extent of the compatibility between the drug and micelle core.

### 3.2. The Core–Drug Compatibility

The compatibility between a polymer and a drug refers to the miscibility and/or interaction with no alteration in the chemical structure of the polymer or the drug [87]. Because each drug has its own unique physicochemical properties, no delivery vehicle prepared from a particular polymer will serve as a universal carrier for all drugs. The degree of compatibility between a polymer and a drug may help in the design of polymeric micelles as delivery systems [29,131]. The interaction strength between the core-forming blocks and the incorporated drug molecules can be estimated by a dimensionless energy parameter called the Flory–Huggins interaction parameter (χ):

(2) $$\chi \;=\;{\left(\delta_{d}-\delta_{lp}\right)}^{2}\frac{V_{m}}{RT}$$

where *δ_d_* and *δ_lp_* are the total solubility parameters of the drug molecules and the core-forming block, respectively, *υ_m_* is the molar volume of the drug, *R* is the universal gas constant, and *T* is the absolute temperature. The lower the interaction parameter, the higher the compatibility between the drug molecules and core-forming blocks, thus leading to enhanced solubilization, as well as slower drug release. This is in accordance with the general rule that chemical and structural similarity favors solubility [132]. The solubility parameter is the square root of the cohesive energy density (*E_coh_*) of the amorphous polymer at room temperature, which is a sum of all forces, including van der Waals dispersion (*E_d_*), dipole–dipole (*E_p_*) interaction, and hydrogen bond (*E_h_*) interaction:

(3) $$E_{coh}\;=\;E_{d}+E_{p}+E_{h}$$

The corresponding equation for the solubility parameter is:

(4) $$\delta_{t}^{2}\;=\;\delta_{d}^{2}+\delta_{p}^{2}+\delta_{h}^{2}$$

Unfortunately, there is no direct way to estimate the partial solubility parameters *δ_d_*, *δ_p_*, and *δ_h_*. Instead, indirect methods can be used to calculate them, including Fedors [133], Hoy [134], Hansen [135], and Hoftyzer-Krevelen [132] methods. However, the experimental data of the solubility parameters for some polymers showed large variations, and the predicted values according to each of the aforementioned methods fall within the experimental limits of accuracy. Therefore, the methods of Hoy and Hoftyzer-van Krevelen are superior to the other methods, and each of them predicts the solubility parameters with a mean accuracy of about 10% [132].

Recently, few studies have appeared on the prediction of the degree of compatibility (interaction) between the polymer and drug systems based on the comparison between the total solubility parameters for both the drug (*δ_d_*) and core-forming blocks (*δ_lp_*) [72,87,136]. The authors considered that the smallest difference between *δ_d_* and *δ_lp_* is an indication of better compatibility between drug and polymer. Based on this suggestion, Liu et al. found an agreement between the theoretical and experimental trend of the preferential solubilization of Ellipticine in different polymers [87]. In contrast, Richter et al. found that the calculated solubility parameters were not predictive, because they showed a reversed order of preference toward Sagopilone solubilization relative to the experimental data [72]. Such discrepancy may arise from the methods used to calculate the solubility parameters, where Liu et al. used the Hansen method to estimate the solubility parameters and the Fedros method to calculate the molar volumes of the drug and polymers. On the other hand, Richter et al. [72] used Software developed by Computer Chemistry Consultancy (Singen, Germany) based on the Hoy method to estimate the solubility parameters [137]. According to the study of van Krevelen and Nijenhuis [132], the Hoy method should give a more accurate estimation for the parameters than the Hansen method, which is not the case if we compare between the results presented in [72,87]. The molar volume of the drug molecules is likely to have a significant impact on these results because Ellipticine (*υ_m_* = 229.97 cm^3^ mol^−1^) has a smaller molar volume than Sagopilone (*υ_m_* = 510.20 cm^3^ mol^−1^). Consequently, a negligible influence on the overall trend of the comparison between the solubility parameters has been shown in case of Ellipticine, whereas the major influence of Sagopilone’s molar volume led to a discrepancy between the experimental results and the theoretical prediction based on the comparison between solubility parameters. Recently, Letchford et al. [66] used the Flory–Huggins interaction parameter to predict the compatibility between the blocks and five different drug molecules. The authors used the Fedors and van Krevelen methods to estimate the molar volume of the drugs and total solubility parameters, respectively. An excellent agreement between the experimental findings and the theoretical prediction (from χ values) for the preferential solubilization (compatibility) of five hydrophobic drug molecules with PCL has been found [66].

The majority of the studies on loading hydrophobic drugs in polymeric micelles were not based on prior predictions of the compatibility between the drug molecules and the core-forming blocks. We can see from Table 3 that PEG_5000_-PCL_5000_ micelles have preferential solubilization for various drug molecules following this trend: Saglopin > Cucurbitacin B > CsA > Paclitaxel > Doxorubicin. PEG_5000_-PBCL_4700_ micelles were found to perfectly solubilize Cucurbitacin B with a loading efficiency (LE) of 92.9% [67], and PEG_2000_-PVL_2000_ micelles were preferred for Paclitaxel, showing a LE of 92% [106]. Therefore, the predication of the drug–polymer compatibility based on the Flory–Huggins interaction parameter χ Equation (1) is presumably more accurate, because the molar volume is included in the equation. Using this method, the prior prediction of the drug–polymer compatibility and its comparison with the experimental findings are shown in the next paragraph.

In an attempt to present the importance of the prior prediction of the core–drug compatibility, we present theoretical calculations of the Flory–Huggins interaction (χ) for a series of biodegradable core-forming blocks (PCL, PBLA, PDLLA, PBLG, PVL, and PLGA) and common water-insoluble anticancer drugs (Paclitaxel (PTX), Camptothecin (CPT), Curcumin (CUR), anti-inflammatory Indomethacine (INN), immunosuppressive Cyclosporin A (CsA), and hypolipidemic (reducing triglyceride and cholesterol concentration in plasma) Fenofibrate (FNB)). For each block and drug molecule, the solubility parameters of the hydrophobic block (*δ_lp_*) and drug (*δ_d_*) are calculated on the basis of the group contribution method by Hoy, using solubility parameter software provided by Computer Chemistry Consultancy (Singen, Germany). The molar volume of the drug molecules (*v_m_*) were calculated using the online Molinspiration calculator based on van der Waals molecule volume [136]. Afterward, the Flory–Huggins interaction parameters for each polymer and drug were calculated using Equation (2). 

**Figure 3:**
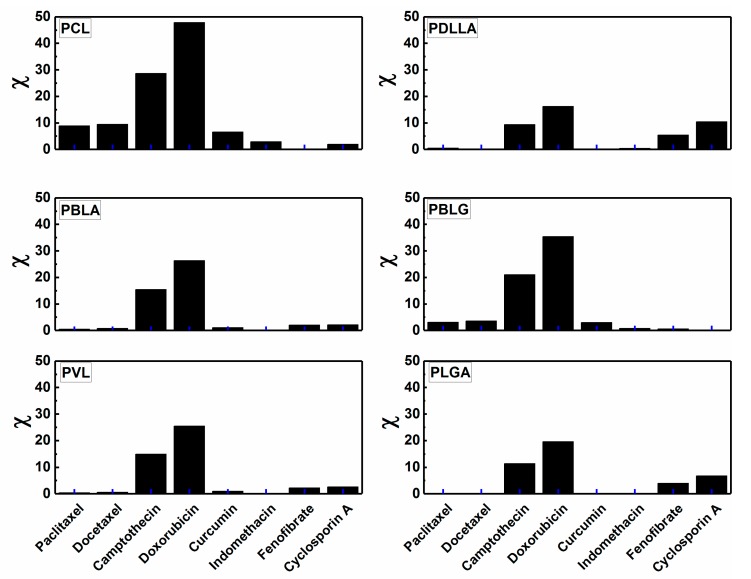
Examples of the calculated Flory–Huggins interaction parameters (χ) for sets of hydrophobic blocks and drugs at 25 °C.

Figure 3 depicts the change in drug–polymer compatibility, expressed by the Flory-Huggins interaction parameter (χ) at 25 °C. Slightly lower χ values are expected at 37 °C. It can be seen that χ values of PCL-drugs are relatively higher than those of other sets of core-forming blocks and drugs. This implies that PCL is not the optimal core-forming block, which is expected to exhibit high loading efficiency, for the majority of the studied drug molecules. It only shows very low χ values with fenofibrate, indicating that PCL is likely to efficiently encapsulate and probably retard the release of this drug. In comparison, the micelles of PDLLA, PBLA, PLGA, and PVL are likely to show much higher encapsulation efficiency for Paclitaxel and Docetaxel (commonly used anticancer drugs) than PCL-based micelles. This is can be viewed from the very low χ values of these hydrophobic blocks with Paclitaxel and Docetaxel. In addition, Camptothecin and Doxorubicin can be more efficiently loaded in PDLLA-based polymeric micelles in comparison with other polymeric micelles.

Smaller the χ value, the more compatible the drug with the core-forming block and hence, the higher loading efficiency. Table 6 demonstrates the χ values for some selected hydrophobic blocks and drug molecules. For example, Cyclosporine A is expected to be efficiently encapsulated in PEG-PBLG micelles in much higher rates than in other polymeric micelles. The maximum LE% for Cyclosporine A was found to be 63.8% in PEG-PCL micelles [51]. Indeed, PEG-PVL micelles have been found to encapsulate 92% of Paclitaxel [99], whereas PEG-PDLLA and PEG-PCL micelles exhibited LE of 25% [36] and 38% [89], respectively. These experimental findings are in accordance with our theoretical predictions, where the Flory–Huggins interaction parameter increases in the following sequence: PVL-PTX < PDLLA-PTX *<<* PCL-PTX. Moreover, it is speculated that PEG-PLGA micelles will exhibit higher LE than 92% due to χ_PLGA-PTX_ < χ_PVL-PTX_, as illustrated in Table 6. Based on these data, we shall revisit the proposed polymer–drug systems in order to better understand the encapsulation efficiency, confirming the strong effect of the interaction between the micelle core and drug molecules at a fixed polymer composition (MW, hydrophobic/hydrophilic ratio).

### 3.3. The Drug/Polymer Ratio

The size and polydispersity of loaded micelles, as well as the loading efficiency, depend on the drug/polymer weight ratio [47,48,86]. There is an optimum ratio above which the micelles are unable to take up any more drug molecules, and then, the drug (and presumably polymer) precipitates, resulting in a decrease in the loading efficiency [59,66]. The influence of the drug/polymer ratio can be clearly assessed over a sufficiently large range. Aliabadi et al. reported an independency of micelle size and loading efficiency on the drug/polymer weight ratio in MPEG-PCL/CsA micelles prepared by cosolvent evaporation method [97]. The loading efficiency of Lidocaine almost remained constant (18–20%) when the Lidocaine/polymer (PEG_5000_-PDLLA_45000_) weight ratios varied from 0.2 to 0.5, whereas at ratio of 1, it was not possible to estimate the aggregate size and loading efficiency [74]. Yokoyama et al. found a decrease in loaded micelle size and loading efficiency as the initial drug concentration increases in a PEG-PBLA/KRN system [19]. The polymeric micelles of PEG_23000_-PCL_45000_ showed a continuous increase in the 17β-Estradiol-loading efficiency (from 36% to 96%) when the 17β-Estradiol/polymer increased from 0.1 to 2 *w*/*w* [98]. Hagan et al. reported this trend for both testosterone and Sudan black B in PEG-PDLA micelles [138]. When the Ellipticine/polymer weight ratio changed from 0.05 to 0.5, the size of the PEG_5000_-PCL_4000_ micelle showed a negligible change (20–24 nm), and the loading efficiency decreased from 72% to 65%, whereas, the size of the PEG_5000_-PDLLA_4000_ micelle strongly increased from 66 nm to 115 nm [87]. The same trend was exhibited by a Honokiol/MPEG-PCL system [99], where a critical drug/polymer ratio of 8/20 was recorded, below which a negligible change in micelle size (29–31 nm) and a decrease in Honokiol-loading efficiency (from 95.8% to 65.4%) were observed. Then, at a drug/polymer ratio of 12/20, larger micelles of 165 nm were formed. 

### 3.4. The Drug Release Kinetics

The kinetics of drug release from polymeric micelles is highly influenced by many factors, including micelle size, length, crystallinity, and polarity of the hydrophobic block and the compatibility between the micelle core and drug molecules. The larger micelle size, the slower the drug release rate [86]. Longer hydrophobic blocks induce slower drug release rate. The drug has further to diffuse in a core with a longer hydrophobic block. A longer core block would also have a higher glass transition temperature [106], so that closer to room temperature, the higher viscosity of the medium would result in a slower release. Finally, the larger core diameter could result in a higher crystallinity of the core in comparison to a smaller core diameter; the higher crystallinity would slow the release of the drug [98]. PEG-PBLG micelles with longer PBLG showed slower drug release rates compared with micelles with shorter hydrophobic blocks, as a consequence of an increased hydrophobic interaction between the drug molecules and PBLG [103]. After 24 h, PEG_5000_-PCL micelles (with PCL/PEG = 0.32–1.0) released only 5% of their Saglopine contents compared with PEG_2000_-PCL with PCL/PEG = 0.7 and PCL/PEG = 1.3, which released 9% and 7%, respectively [72]. 17β-Estradiol was released faster from PEG_1980_-PCL_2622_ than from PEG_1980_-PCL_17328_, because the latter micelle presumably has a larger core due to the longer PCL so that the 17β-Estradiol has further to diffuse in a core with a longer hydrophobic block [98]. In contrast, it has been found that the length of the hydrophobic block has no significant effect on the release rate; instead, the higher the hydrophobicity, the slower the release rate [64,65]. A longer core block would also have a higher glass transition temperature, so that closer to room temperature, the higher viscosity of the medium would result in a slower release. Over two weeks, the loaded Ellipticine was released more slowly from the PEG-PBLA micelle (1.4%) than from the PEG-PCL micelle (5.4%) under the sink conditions [87]. This behavior is attributed to the fact that a greater degree of interaction between the polymer and the drug leads to a slower drug release. In the same study, the authors found that the rate of drug release from the micelles decreased with an increase in the drug/polymer ratio at constant copolymer concentration. In different study, PEG-PCL micelles were found to completely release the loaded FK506 after six days [94]. Doxorubicin-loaded MPEG-PCL micelles showed a faster drug release at pH 5 (<80% over a month) than pH 7 (<20% over a month) [96]. This faster release of Doxorubicin in an acidic medium was also observed by Kataoka and coworkers [105] with the Doxorubicin-loaded PEG-PBLA micelles and is likely due to the re-protonation of the amino group of Doxorubicin and the faster degradation of the micelle core at a lower pH. This pH-dependent releasing behavior is of particular interest in achieving the tumor-targeted Doxorubicin delivery with micelles. La et al. [10] have found that Indomethacin more rapidly released from PEG-PBLA micelles in an alkaline medium than from an acidic medium. The authors explained this behavior on the basis that the release is controlled by the partition coefficient of the drug based on the pH of the medium and the hydrophobic–hydrophobic interaction between the drug and the hydrophobic core of the micelles. Independent of the amount of loaded Paclitaxel, MPEG-PCL micelles showed high stability against dilution in water, with less stability in a buffer solution of pH 10 and in serum albumin [96]. Moreover, no size changes were detected over two weeks; however, after three months, larger aggregates or even precipitates were observed especially for micelles with longer PCL, independent of the amount of loaded Paclitaxel. MPEG-PCL micelles efficiently presented higher loading efficiency and more sustained drug release than commercial Cremophor EL micelles [97]. Within 12 h, 5.8% of CsA was released in vitro from MPEG-PCL micelles, while Cremphor EL micelles released 77% of their loaded drug, implying the higher viscosity of the polymeric micelle cores. It is worth mentioning that the control of micelle dissociation and the drug release rate is essential for drug targeting and that this control of these matters is sometimes technically difficult to optimize for such targeting, although this is not a disadvantage of the polymeric micelle systems [76].

Drug release is highly influenced by where the drug molecules are located [49]. If the drug is located predominantly in the corona, then the length of the core-forming block, the micelle size, and the molecular volume of the drug are less important in determining the release rate. Gorshkova and Stotskaya [139] observed a faster release of Daunomycin when the micelle has smaller PEG units. On the other hand, the amount of drug loaded in the micelle core is the determining factor for the release rate if the drug molecules are predominantly located in the core; the higher the concentration of drug, the slower the release rate. Jeong et al. [103,104] have shown that the release of both Adriamycin and Clonazepam from PEG-PBLG micelles is slower for higher concentrations of the respective drugs. At low loadings, Gref et al. also observed that Lidocaine was molecularly dispersed in the hydrophobic cores of the PEG-PLGA micelles, resulting in a faster release [101]. At high loadings, they showed that the release of Lidocaine was slower because of possible drug crystallinity. Similarly, at high loadings of Lidocaine, Görner et al. [86] observed crystallinity of the drug in PDLLA micelles. Crystallinity of the drug slows the release, because release from the particles is possible only after the crystallized drug has dissolved and diffused to the outer solution [59].

### 3.5. The Micelles Preparation and Drug-Loading Methods

The physical loading efficiencies of the drug molecules in polymeric micelles were found to be dependent on the incorporation methods [72,97,140,141]. Table 3 demonstrates the effect of the preparation method on the micelle size and the drug-loading efficiency. Sonication turned out to be a very effective method for dramatically reducing the aggregate size, much more than centrifugation and extrusion [87], depending on the sonication time [87,141] and the nature of the block copolymer, where larger and denser aggregates were formed upon sonication of triblock-based micelles [141]. Both centrifugation and extrusion were found to decrease strongly the amount of loaded drug [111,142]. Kwon et al. found that heating enhances the incorporation of pyrene molecules (as a drug model) in PEO-PBLA polymeric micelles, much more than stirring or sonication [40]. In addition, a clear micellar solution was obtained after minutes of sonication, whereas an overnight equilibration is needed to obtain a clear solution after stirring. Görner et al. found that smaller micelles were obtained when low-phase (organic and aqueous) volume ratios and high surfactant concentration were used during the preparation of PEG-PDLLA micelles by the emulsion-solvent evaporation method [86].

Micelle size and stability and the loading efficiency are highly dependent on the solvent used in the preparation method. Shin et al. prepared drug-loaded micelles of 156 nm with higher loading efficiency (42.2%) when dimethylformamide (DMF) was used in the dialysis method, and a tetrahydrofuran (THF)-based loading method gave 165 nm with 17.73% at the same conditions [47]. Significant variation of the micelle size (from 114 to 181 nm) and size distribution was noticed when using four different solvents to prepare MPEG-PCL micelles using dialysis [48]. In the preparation of PEG-PBLA micelles by dialysis, very large micelles (ca. 300 nm) [111] or secondary aggregates were formed when using dimethyl sulfoxide (DMSO), and only 6% of the total copolymers were micellized, [10] whereas smaller micelles (100–200 nm) were formed with DMF [111], and much smaller ones (ca. 19 nm, with PDI = 1.27) were obtained when dimethylacetamide (DMAc) [10] was used during preparation. Among different solvents (chloroform, methylene chloride, ethyl acetate, acetone, methanol, ethanol, THF, and acetonitrile) used to prepare Taxol-loaded polymeric micelles of MPEG-PDLLA, acetonitrile was the only solvent that produced a clear micellar solution [45]. Aliabadi et al. demonstrated that the replacement of water with normal saline (to prepare isotonic polymeric micellar solutions of CsA for intravenous administration) did not affect the average diameter of unloaded and CsA-loaded MPEG-PCL micelles prepared by the solvent-evaporation method [61,97]. However, the drug-loading efficiency was reduced in normal saline solution because of a premature precipitation of the drug during the micellization process. No systematic effect of the solvent has been noticed in the PEG-PBLA/KRN 5500 system, because both DMF and DMSO offered higher drug loading at a particular drug/polymer ratio for each solvent [111,142]. Recently, Harada et al. have shown significant differences in the drug-incorporation behaviors in the morphologies of the incorporated drug and the polymeric micelles and in the pharmacokinetic behaviors when using two solvents (trifluoroethyl alcohol and chloroform) in the solvent-evaporation method to load Camptothecin in polymeric micelles [136].

Recently, Richter et al. reported an interesting correlation between the preparation method, the hydrophobic/hydrophilic ratio, and the Sagopilone loading efficiency in the polymeric micelles of PEG-PCL and PEG-PDLLA [72]. In general, the sonication method was more appropriate for preparation and achieving higher loading efficiency in the PEG-PCL micelles, whereas, the film-formation method gave much higher loading efficiency with the PEG-PDDLA micelles (see Table 3). The film-formation method seems to appreciably achieve the highest loading efficiency (>90%) only at low hydrophobic/hydrophilic ratios (0.3), and supersaturation effect occurs at higher ratios. This is in contrast to the statement by Aliabadi et al. that film-formation method is not applicable to produce PEG-PCL based micelles [97]. Richter et al. also found that the PEG-PCL micelles prepared by sonication were stable for at least 24 h, in contrast to those prepared by film-formation method [72]. Larger PEG-PCL micelles with lower drug-loading efficiency were formed in absence of ultrasonication, compared with those prepared under ultrasonication [138]. After micelle preparation by the emulsion-solvent evaporation method, filtration resulted in relatively smaller PEG-PVL micelles with lower loading efficiency compared with centrifugation [106].

The effect of the preparation methods strongly influences the micelle size and size distribution (polydispersity) [72,97,140]. As can be seen in Table 3, the preparation method plays a significant role in determining the size and polydispersity of micelles and the drug-loading efficiency, aside from the block copolymer molecular weight. At present, there seems to be no universal preparation/incorporation method applicable to any polymer–drug systems. Therefore, finding an appropriate incorporation method for each drug through trial and error is required. Furthermore, in some methods, the drug incorporation may be difficult on a large industrial scale but easy and efficient on a small laboratory scale [76]. The scale problem is more serious than the polymer synthesis matter, because physical factors (e.g., diffusion and solvent exchange rate) are strongly influenced by the scales in the drug incorporation processes, such as solvent exchange through a dialysis membrane. Therefore, more scientific and engineering studies are necessary for significant development in the incorporation technology.

### 3.6. Real Drug Release Kinetics

There is still a poor understanding of how the micelles release the drug, whether the drug molecules diffuse freely from the intact micelle core or after the bursting of the micelles. Some researchers have reported a biphasic release profile [98]. In vitro release studies have been conducted in a medium simulating physiological conditions in an isotonic buffer solution of pH 7.4 at 37 °C [10,68,86,96,103] in the presence of lipase or proteins [106]. Rationally, the micelle stability, core-drug compatibility, and the molar volume of a drug, as well as the physiological conditions (pH 7.4 and 37 °C) are crucial factors that influence the release kinetics of a drug from the polymeric micelles. Nevertheless, the mechanical forces exerted on the polymeric micelles as a result of shear rates range from ca. 50 s^−1^ (in the veins) to ca. 500 s^−1^ (in the small capillaries) might have strong influences on the drug release rate. So far, no studies have been conducted to investigate this issue, which is highly necessary to more precisely determine drug release rates so that the optimization of drug formulations will be accounted for according to one more factor: *the release under flow, which* leads to understanding the *real drug release kinetics*. 

## 4. Conclusions and Perspectives

It is of significant interest to design polymeric micelles that are capable of acting as true delivery vehicles for various potent drugs, which are not in therapeutic formulations due to their water-insoluble, hydrophobic natures. After demonstrating in detail the factors that govern micellar stability and size, loading efficiency, and drug release kinetics, we can recap these factors briefly as follow: (1) A relatively small number of polymers have been administered in the human body and clinically validated as safe for systemic administration in the body [143]. Examples of such approved polymers are the biocompatible hydrophilic PEG [144] and the biodegradable hydrophobic PLGA [145] and PDLLA [123]. However, we have not restricted our study to these approved polymers, and polymers such as PVL and PBLA have been considered due to their ability to efficiently solubilize hydrophobic drugs, as shown by their high compatibility with some anticancer drugs; (2) Thermodynamically and kinetically stable polymeric micelles in vitro and in vivo (under sever dilution) with very low CMC can be obtained by choosing a block copolymer with a particular MW and hydrophobic/hydrophilic ratio. Block copolymers composed of PEG with a moderate MW (5 kDa) and a hydrophobic/hydrophilic ratio between 0.5 and 1 have been found to form stable micelles with low CMC; (3) This polymer composition produced polymeric micelles with sizes of <100 nm, which overcome recognition by the RES and size-sieving in the bloodstream if they are monodisperse (depending on the polydispersity of polymer and the preparation method); (4) Finally, after fixing this polymer composition, the compatibility between the micelle core and the drug molecules in terms of the Flory–Huggins interaction parameter (χ) is a critical determining factor of the extent of the drug-loading efficiency and kinetic release. The crystallinity of core-forming blocks and the molar volume and partition coefficient of drug molecules may have minor impacts on the loading efficiency and release rate.

In this review, we highlighted the importance of prior prediction of the drug–core compatibility by calculating the Flory–Huggins interaction parameter to optimally select an appropriate core-forming block for a specific anticancer drug. This necessarily aids in the optimization of the design of an ideal polymeric micelle for cancer therapy, taking into account all the biological requirements (biodegradable blocks), increased drug-loading efficiency, and retarded drug release. 

Despite the significant number of studies on drug-loading and targeting using polymeric micelles, however, there still a lack of full understanding of the mechanism of encapsulation in the polymeric micelles and the enhancement of the loading capacity, as well as the stability of the loaded micelles in vivo and in vitro. Comparisons between various copolymer/drug systems with different natures, composition preparation methods, and other parameters lead sometimes to conflicting conclusions about the factors that govern micelle stability and size, drug-loading efficiency, and release kinetics. Accordingly, it is highly important to overcome the following challenges facing the potency of polymeric micelles for drug encapsulation and delivery by resolving the following challenges:

1. Enhancing the drug-loading capacity in polymeric micelles through selecting the appropriate polymer with its hydrophobic core-forming blocks to favorably solubilize many drug molecules based on a prior prediction of the compatibility between the micelle core and a particular drug through the calculation of the Flory–Huggins interaction parameter (χ).
2. Investigation of the effect of the micelle preparation method and solvents used on the micelle size, morphology, polydispersity, and stability, as well as the drug-loading efficiency and release kinetics.
3. The polymer compositions (i.e., the molecular weight and hydrophobic/hydrophilic ratio) should be put in narrow distribution in order to eliminate their effect on micelle size (and hence, the loading efficiency of micelles), as well as to produce micelles of size <100 nm. The drug/polymer ratio should be tuned, because there is no common optimum value that produces the highest drug-loading without polymer and drug precipitations.
4. Beside the interaction strength between the core and the drug, the flow rate in the bloodstream might have strong influence on the drug release rate. This issue has not been studied so far. This is can be investigated in a microfluidic cell to mimic the flow rate in the blood capillaries in order to precisely account for the drug release profile in an environment simulating the blood circulatory system.

## Figures and Tables

**Table 1:** Rational design of an “ideal” polymeric micelle for cancer therapy taking into account all the biological requirements. Adapted with permission from [28].

Requirement	Consequences in Polymeric Micelle Design
Protect drug from degradation	Encapsulation into the micellar core.
Intravenous injection	Sub-100 nm.
Obtain the desired micelle size	Adjust the physicochemical properties of the polymer with its constituents and apply an adequate preparation method.
Prevent opsonization	Coating with hydrophilic polymer (PEG).
Decrease the drug release rate	Adjust the core–drug compatibility (χ).
Control of biodistribution	Introduction of targeting moieties (antibodies, peptides, carbohydrates).
Control of pharmacokinetics and pharmacodynamics	All previous parameters.
Elimination	Use of biocompatible and biodegradable materials.

**Table 2:** Factors affecting the performance-released properties of polymeric micelles as drug carriers. Adapted with permission from [29]. MW: molecular weight; *C*: concentration; HLB: hydrophilic-lipophilic balance; PDI: polydispersity index; *δ_ph_*, *δ_pl_*, *δ_d_*: solubility parameters of hydrophilic, hydrophobic blocks, and drug, respectively; T_g_: glass transition temperature; *v*_m_: the molar volume of the drug molecules; *K_d_*: the dissociation rate; LE: loading efficiency; and RP: release profile.

Factors		Performance Properties					
		Stability, *CMC, K_d_*	Size	Surface Properties, Hydrophilicity	Morphology	LE	RP
**Block copolymer**	MW						
	*C*						
	HLB						
	PDI						
**Core blocks**	*Length*						
	*δ_pl_*						
	T_g_						
	Biodegr-adibility						
**Corona blocks**	*Length*						
	*δ_ph_*						
	Biocomp-atibility						
**Drug**	*δ_d_*						
	*v_m_*						
	*C*						
**Preparation method**							

**Table 3:** Comparative survey of some block copolymers, their characteristics, and their efficiency in encapsulating hydrophobic drugs (loading efficiency, LE%) using different preparation methods.

Polymer	Core/Corona wt Ratio	Method	Size of Unloaded Micelles (nm)	Drug	Size of Loaded Micelles (nm)	LE%	Ref.
PEG_5000_-PCL_5000_	1.0	Sonication	69.0	Saglopine	ND	70	[72]
PEG_2000_-PCL_1400_	0.7		55.0		ND	66	
MPEG_5000_-PCL_5000_	1.0	Cosolvent evaporation	87.5	CsA	100	52.2	[97]
MPEG_5000_-PCL_13000_	2.6		78.7		98.6	63.8	
MPEG_5000_-PCL_24000_	4.8		99.8		102.3	49.5	
PEG_2000_-PCL_2000_	1.0	Solvent displacement/sonication	17.0	Doxorubicin	25.4	3.29	[108]
MPEG_5000_-PCL_2500_	0.5		29.7		22.9	3.10	
MPEG_5000_-PCL_5000_	1.0		41.0		37.3	4.03	
MPEG_5000_-PCL_8500_	1.7		56.9		84.0	4.09	
MPEG_5000_-PCL_24700_	4.9		86.3		104.9	4.30	
MPEG_2000_-PCL_1200_	0.6	Cosolvent evaporation	29.4	Paclitaxel	31.3	3.3	[96]
MPEG_2000_-PCL_2700_	1.4		37.3		42.6	13	
MPEG_5000_-PCL_3800_	0.7		71.8		65.3	23	
MPEG_5000_-PCL_18000_	3.6		97.7		91.9	38	
PEG_5000_-PCL_4000_	0.8	Dialysis	ND	Ellipticine	20 ^a^	75.9 ^a^	[87]
		Dry down	ND		76 ^a^	65.3 ^a^	
PEG_2000_-PCL_900_	0.5	Dialysis	ND	FK506	50	21	[94]
PEG_1980_-PCL_1368_	0.3	Dialysis	ND	17*β*-estradiol	ND	10	[98]
PEG_1980_-PCL_2622_	0.5		25		30	19	
PEG_1980_-PCL_17328_	3.4		ND		ND	90	
PEG_2000_-PCL_2280_	1.1	Cosolvent evaporation	ND	Cabazitaxel	28.8	99.3	[109]
PEG_5000_-PCL_5000_	1.0	Cosolvent evaporation	ND	Cucurbitacin B	73.3	30.2	[67]
PEG_5000_-PCL_24000_	4.8		ND		78.3	65.1	
PEG_5000_-PCL_5000_	1.0		ND	Cucurbitacin I	72.2	44.1	
PEG_5000_-PCL_24000_	4.8		ND		77.2	68.4	
PEG_5000_-PCL_4790_	1.0	Dialysis	62.5	Paclitaxel	69.2	24.7	[100]
PEG_5000_-PCL_10000_	2.0	Cosolvent evaporation	ND	Dasatinib	54.3	95.4	[110]
MPEG_5333_-PCL_2638_	0.5	Dialysis	54	Indomethacin	ND	ND	[47]
MPEG_5333_-PCL_4984_	0.9		77		ND	ND	
MPEG_5333_-PCL_8034_	1.5		114		120–165 ^b^	16.8–42.2 ^b^	
MPEG_5333_-PCL_9068_	1.7		130		ND	ND	
MPEG_5000_-PCL_2166_	0.4	Emulsion-solvent evaporation	45.3	Indomethacin, Curcumin, Plumbagin, Paclitaxel, Etoposide	See Ref. [66]		[66]
MPEG_2000_-PCL_1320_	0.7		22.3				
MPEG_2000_-PCL_852_	0.4		14.7				
MPEG_750_-PCL_464_	0.6		12.4				
MPEG_750_-PCL_323_	0.4		13.5				
MPEG_750_-PCL_197_	0.3		11.1				
MPEG-PCL		Direct dissolution assisted by ultrasound	27	Honokiol	31 ^c^	65.4 ^c^	[99]
PEG_5000_-PDLLA_4200_	0.8	Dialysis	ND	Ellipticine	76	1.2	[87]
		Dry down	ND		106	6.2	
PEG_5000_-PDLLA_45000_	9.0	Emulsion-solvent evaporation	ND	Lidocaine	203	17	[86]
MPEG_2000_-PDLLA_2000_	1.0	Emulsion-solvent evaporation	ND	Paclitaxel	<50 nm	25	[45]
MPEG_2000_-PDLLA_1333_	0.7		ND			25	
MPEG_5000_-PDLLA_2143_	0.4		ND			10	
PEG_52000_-PDLLA_56000_	1.1	Dialysis	33	ND	ND	ND	[91]
PEG_91000_-PDLLA_56000_	0.6		30		ND	ND	
PEG_4100_-PDLLA_1200_	0.3	Dialysis	154	ND	ND	ND	[35]
PEG_6000_-PDLLA_3000_	0.5		28.1		ND	ND	
PEG_5700_-PDLLA_5400_	1.0		33.5		ND	ND	
PEG_6100_-PDLLA_7800_	1.3		35.0		ND	ND	
PEG_5000_-PBCL_4700_	0.9	Cosolvent evaporation	ND	Cucurbitacin B	76.3	92.9	[67]
			ND	Cucurbitacin B	74.1	74.1	
PEG_5000_-PBCL_4470_	0.9	Dialysis	64.3	Paclitaxel	61.0	36.4	[100]
PEG_12000_-PBLA_5000_	0.4	Dialysis ^d^	19	Indomethacin	29	20.4	[10]
		o/w emulsion	ND		25	22.1	
PEG_12000_-PBLA_3000_	0.3	Dialysis	20	Amphotericin B	25.8	27–30 ^e^	[84]
PEG-PBLA		Dialysis	>100	KRN 5500			[111]
PEG_12000_-PBLA_5000_	0.4	o/w emulsion	19	Doxorubicin	37	65	[43]
MPEG_2000_-PVL_1000_	0.5	Emulsion-solvent evaporation	ND	Paclitaxel	200	37	[106]
MPEG_2000_-PVL_2000_	1.0		ND		31	92	
MPEG_5000_-PVL_2600_	0.5		ND		225	10	
MPEG_5000_-PVL_4900_	1.0		ND		138	3	

^a^ These values were taken at drug/polymer = 1/10. ^b^ These values depend on the drug/polymer weight ratio and the solvent used in the dialysis method. ^c^ The drug-loaded micelle size and loading efficiency at drug/micelle = 8/20. ^d^ Dialysis method was used to prepare unloaded micelles, and dialysis and *o*/*w* emulsion methods were used to prepare drug-loaded micelles. ^e^ The drug-loading efficiency ranges from 27 to 30%, depending on the drug/polymer ratio. ND = not determined.

**Table 4:** Variation of the CMC of various block copolymers with the hydrophobic/hydrophilic ratio and hydrophobic length.

Polymer	Core/Corona Ratio	CMC (mg/L)	Ref.
PEG_5000_-PCL_5000_	1.0	1.8	[97]
PEG_5000_-PCL_13000_	2.6	0.8	
PEG_5000_-PCL_24000_	4.8	0.5	
MPEG_5000_-PCL_2166_	0.4	4.5	[66]
MPEG_2000_-PCL_852_	0.4	21.0	
MPEG_750_-PCL_323_	0.4	122.1	
MPEG_750_-PCL_464_	0.6	71.5	
PEG_12000_-PBLG_8400_	0.7	2.7	[103]
PEG_12000_-PBLG_39800_	3.3	2.2	
PEG_12000_-PBLG_91700_	7.6	2.0	
PEG_5000_-PBLA_2381_	0.5	10	[12]
PEG_5000_-PBLA_4762_	1.0	5	
PEG_12000_-PBLA_4762_	0.4	10	
PEG_12000_-PBLA_5000_	0.4	18	[10]
PEG_6000_-PDLLA_3000_	0.5	4.5	[35]
PEG_6100_-PDLLA_7800_	1.3	2.5	
MPEG_2000_-PVL_550_	0.3	176	[106]
MPEG_2000_-PVL_1000_	0.5	80.4	
MPEG_2000_-PVL_2000_	1.0	23.3	

**Table 5:** Common core-forming blocks and their characteristic crystallinity and glass transition temperatures (T_g_).

Polymer	T_g_ (°C)	State	Ref.
PCL	−60	Semicrystalline	[128]
PVL	−47 to −70	Semicrystalline	[106]
PDLLA	34.5	Amorphous	[35]
PLGA	40–60	Amorphous	[129]
PBLA	-	Amorphous	[87]
PBLG	50	-	[130]

**Table 6:** The calculated Flory–Huggins interaction parameters (χ) for different block copolymers and hydrophobic drug molecules at 25 °C.

Drug	χ_PCL-drug_	χ_PDLLA-drug_	χ_PBLG-drug_	χ_PVL-drug_	χ_PLGA-drug_
Fenofibrate	0.002	5.380	0.563	2.222	3.983
Curcumin	6.563	0.017	3.047	0.969	0.216
Cyclosporine A	1.942	10.455	0.026	2.591	6.756
Indomethacin	2.872	0.422	0.823	0.030	0.108
Paclitaxel	8.908	0.470	3.069	0.363	0.033
Camptothecin	28.691	9.338	21.011	14.924	11.365
